# Spatially resolved characterization of tissue metabolic compartments in fasted and high-fat diet livers

**DOI:** 10.1371/journal.pone.0261803

**Published:** 2022-09-06

**Authors:** Sylwia A. Stopka, Jiska van der Reest, Walid M. Abdelmoula, Daniela F. Ruiz, Shakchhi Joshi, Alison E. Ringel, Marcia C. Haigis, Nathalie Y. R. Agar

**Affiliations:** 1 Department of Neurosurgery, Brigham and Women’s Hospital, Harvard Medical School, Boston, MA, United Statees of America; 2 Department of Radiology, Brigham and Women’s Hospital, Harvard Medical School, Boston, MA, United Statees of America; 3 Department of Cell Biology, Blavatnik Institute, Ludwig Center, Harvard Medical School, Boston, MA, United Statees of America; 4 Bouvé College of Health Sciences, Northeastern University, Boston, MA, United Statees of America; 5 Department of Cancer Biology, Dana-Farber Cancer Institute, Boston, MA, United Statees of America; University of Florida, UNITED STATES

## Abstract

Cells adapt their metabolism to physiological stimuli, and metabolic heterogeneity exists between cell types, within tissues, and subcellular compartments. The liver plays an essential role in maintaining whole-body metabolic homeostasis and is structurally defined by metabolic zones. These zones are well-understood on the transcriptomic level, but have not been comprehensively characterized on the metabolomic level. Mass spectrometry imaging (MSI) can be used to map hundreds of metabolites directly from a tissue section, offering an important advance to investigate metabolic heterogeneity in tissues compared to extraction-based metabolomics methods that analyze tissue metabolite profiles in bulk. We established a workflow for the preparation of tissue specimens for matrix-assisted laser desorption/ionization (MALDI) MSI that can be implemented to achieve broad coverage of central carbon, nucleotide, and lipid metabolism pathways. Herein, we used this approach to visualize the effect of nutrient stress and excess on liver metabolism. Our data revealed a highly organized metabolic tissue compartmentalization in livers, which becomes disrupted under high fat diet. Fasting caused changes in the abundance of several metabolites, including increased levels of fatty acids and TCA intermediates while fatty livers had higher levels of purine and pentose phosphate-related metabolites, which generate reducing equivalents to counteract oxidative stress. This spatially conserved approach allowed the visualization of liver metabolic compartmentalization at 30 μm pixel resolution and can be applied more broadly to yield new insights into metabolic heterogeneity *in vivo*.

## Introduction

Advances in single-cell analysis approaches have revealed that cells within tissues can be metabolically distinct and have unique contributions to physiology and pathology [1]. Metabolic compartmentalization between cellular organelles, within organs, and at the whole-body level is essential to meet the bioenergetic and anabolic demands of organisms. Additionally, metabolically distinct microenvironments develop within tissues based on physiological factors such as proximity to the vasculature, which supplies nutrients and oxygen while removing metabolic waste products.

The liver is organized by regions of functional and spatial heterogeneity. Hepatocytes are structured in neat rows along the liver lobule axis from the portal vein that receives venous blood from the gut towards the central vein, which returns the blood into circulation [2]. As such, the oxygen gradient is highest for periportal hepatocytes and decreases towards the pericentral area [3]. Opposing gradients of oxygen and Wnt signaling along with the radial lobule axis drive differential gene expression signatures[4]: approximately half of all genes in mouse hepatocytes are expressed in a zonated fashion in both space and time [5–7]. This organization drives profound differences in metabolism: periportal hepatocytes rely on the oxidation of fatty acids for energy and perform metabolic functions such as gluconeogenesis, the urea cycle, and biosynthesis of cholesterol and proteins [8]. In contrast, pericentral hepatocytes display glycolytic energy metabolism and synthesize lipids, bile, and glutamine.

The liver plays an essential role in maintaining whole-body metabolic homeostasis in response to nutrient abundance and restriction [9]. In a satiated state, hepatocytes oxidize glucose to generate energy and synthesize fatty acids [10]. Fatty acids are then esterified into triacylglycerols (TAGs) and transported to the adipose tissue for storage. In fasted conditions, the adipose tissue releases fatty acids for oxidation by the liver to yield ketone bodies that can fuel distant organs [11]. Additionally, the liver performs glycogenolysis and gluconeogenesis to restore circulating glucose levels upon fasting. In contrast, upon prolonged nutrient excess conditions, the liver acts as an overflow depot for lipids when the endocrine and storage functions of the adipose tissue become compromised [12]. With rising rates of obesity, nonalcoholic fatty liver disease (NAFLD) is an increasing cause of morbidity and mortality.

Despite the liver’s central role in metabolic homeostasis, liver metabolism is characterized mostly on the gene, protein, and signaling levels. However, as hepatocytes make up over 80% of liver mass [13], metabolite profiles obtained with conventional extraction-based metabolomic methods skew towards hepatocellular metabolism at the expense of other resident cell types. Hepatic heterogeneity can be investigated by mapping many key enzymes using immunohistochemistry. For example, higher levels of glucose-6-phosphatase, succinate dehydrogenase, and phosphoenolpyruvate carboxykinase were expressed in the periportal compared to the centrilobular cells where lactate dehydrogenase, glutamate dehydrogenase are highly expressed [14,15]. Spatially resolved metabolite profiling could yield new insights into metabolic heterogeneity and functionally specialized regions within the liver.

Matrix-assisted laser desorption/ionization (MALDI) mass spectrometry imaging (MSI) is a label-free technique that allows for *in situ* spatial mapping and quantification of hundreds of metabolites from a single tissue section [16–18]. Recent mass spectrometric advances have led to an increasingly higher spatial resolution that now approximates single-cell and sub-cellular analytic capability [19,20]. However, several outstanding challenges in sample preparation and data acquisition needs to be addressed to ensure the robustness of metabolome-scale analyses [21,22]. Unique adaptations are required to yield reproducible and biologically relevant data for small metabolite analyses, including quenching metabolic activity, metabolite stabilization, matrix optimization, and data acquisition [16]. Several studies investigating some aspects of liver metabolism using MALDI MSI have been published, including lipid analysis, bile acid metabolism, N-glycans, drug metabolism and protein distributions [14,23–25].

In this study, we implemented MALDI MSI to spatially map the distribution of small metabolites to recapitulate key bioenergetic activities. We interrogated the liver metabolic response to nutrient stress and excess conditions with a spatial resolution of identified patterns of metabolic specialization within liver tissues. We observed that fasting induced metabolic shifts in central carbon metabolism in the liver in a spatial manner, while in conditions of prolonged nutrient excess induced by a high-fat diet, mice develop fatty livers that remodel central carbon metabolism towards increased pentose phosphate pathway and purine metabolism. Taken together, we show that introducing spatiality into metabolomic analyses reveals an additional layer of metabolic complexity and that our workflow can be applied broadly to yield new insights into metabolic heterogeneity *in vivo*.

## Materials and methods

### Mouse studies

C57BL/6J (000664) and BALB/cJ (000651) mice were obtained from The Jackson Laboratory. Mice were housed at 20–22°C on a 12 h light/dark cycle with ad libitum access to food (PicoLab Rodent Diet 5053) and water. All animal studies were performed in accordance with Haigis lab protocols approved by the Standing Committee on Animals, the Institutional Animal Care and Use Committee at Harvard Medical School. For heat inactivation studies, 3 mice were used (C57BL/6J, female, 7 weeks old) and kidneys, brain halves, and liver lobes from the same individual animal were subjected to the different heat inactivation treatments (overview in S1A and S1E Fig). For desiccation experiments, 2 mice were used (C57BL/6J, male, 7 weeks old). For fasting experiments, two independent cohorts of 5 mice were used per treatment group (BALB/cJ, female, 10–11 weeks old) and mice were subjected to a 16 hour overnight fast. For HFD experiments, two independent cohorts of 4 mice were used per treatment group (C57BL/6J, female). Mice were assigned at 5 weeks old to the control diet (PicoLab Rodent Diet 5053) or HFD (Research Diets, Inc. #12492) and maintained on this diet for 4.5 months. The control diet is 4.07 Gross Energy Kcal/g. The HFD is 5.21 Kcal/g. for 8–10 weeks. Comparative MALDI MSI and LC-MS analyses of tissues were always performed on the same tissue specimens.

### Tissue isolation

Mice were anesthetized with isoflurane and sacrificed by cervical dislocation. The gall bladder was removed before livers, kidneys, and brains were harvested and carefully positioned into 15 mL flat bottom specimen vials (Nalgene, Millipore Sigma), snap-frozen in liquid nitrogen, and stored at -80°C until further processing. The right liver lobe was used for all analyses.

### Tissue heat inactivation

Freshly resected or snap-frozen tissues were placed in sealed Maintainor® tissue cards and placed in the Stabilizor™ system (Denator AB, Gothenburg, Sweden). Sample state was specified (frozen or fresh) and the instrument determined durations of heat treatment based on sample volume for consistent and reproducible heat treatment, according to the manufacturers instructions. Next, tissues were carefully positioned into 15 mL flat bottom specimen vials (Nalgene, Millipore Sigma), snap-frozen in liquid nitrogen, and stored at -80°C until further processing.

### Tissue preparation for MALDI MSI

Frozen tissues were placed at -20°C before sectioning in a Microm HM550 cryostat (Thermo Scientific™). Tissues were sectioned at 10 μm thickness and thaw mounted onto indium-tin-oxide (ITO)-coated slides (Bruker Daltonics) for MALDI MSI analysis with serial sections mounted onto glass slides for histological analyses. The microtome chamber and specimen holder were maintained between -15°C and -20°C. Slides were stored at -80°C until further processing. For desiccation experiments, slides were subjected to desiccation in a tabletop vacuum desiccator before freezing.

### Matrix deposition

A 1,5-Diaminonaphthalene(DAN)-HCl matrix solution was used for all experiments. To generate the hydrochloride derivative of 1,5-DAN, 39.5 mg of 1,5-DAN was dissolved in 500 μL of 1 mol/L hydrochloride solution with 4 mL HPLC-grade water. The solution was sonicated for 20 minutes to dissolve 1,5-DAN, after which 4.5 mL ethanol was added to yield the matrix solution. Matrices were deposited on slides and tissues using a TM-sprayer (HTX imaging, Carrboro, NC). DAN-HCl matrix spray conditions used where: a flow rate of 0.09 mL/min, spray nozzle temperature of 75°C, and spray nozzle velocity of 1200 mm/min. A four-pass cycle was used with 2 mm track spacing and the nitrogen gas pressure was maintained at 10 psi.

### MALDI MSI data acquisition

A timsTOF fleX mass spectrometer (Bruker Daltonics) was used for data collection, and data was acquired using FlexImaging 5.1 software (Bruker Daltonics). The instrument was operated in negative ion mode covering the *m/z* range of 100–1350 for heat inactivation experiments and 100–1250 for desiccation experiments; a spatial resolution of 50 μm was used to define a pixel. The mass resolution of the MALDI MSI method was 35,500 at *m/z* 346 and 34,000 at *m/z* 505. For measurements of metabolites in tissue from fasting experiments, the instrument was operated in negative ion mode covering the *m/z* range of 50–1000; a spatial resolution of 30 μm was used to define a pixel. Each pixel consisted of 800 laser shots, in which the laser frequency was set to 10,000 Hz. A mixture of ^15^N_5_-ATP (10 μM), ^15^N_5_-AMP (1 μM), and ^15^N_-_glutamate (100 μM) was spiked into the matrix and used to calibrate the mass range.

### MALDI MSI data analysis

MSI data were analyzed and visualized using SCiLS Lab 2021a software (Bruker Daltonics). Imported peaks were converted to local max (centroid data) using the mean spectra with a minimal interval width of 5 mDa. Peaks were normalized to total ion current (TIC). Ion images for metabolites of interest were generated based on peak lists containing theoretical *m/z* and an experimental tolerance threshold < 5 ppm from the theoretical values. To generate segmentation maps showing regions of spectral similarity, bisecting *k*-means clustering was applied to all individual peaks in the dataset using the correlation distance metric in SCiLS Lab 2021a software. Intravascular and extravascular ROIs were selected within a tissue boundary limit of 180 μm to avoid edge artifact signal effects. Vascular regions were defined based on the distribution of heme B, and tissue regions based on the segmentation maps, and regions of interest (ROIs) were drawn by hand. For feature annotations and statistical analyses; ROIs defined in SCiLS lab were exported to MetaboScape 6.0 (Bruker Daltonics). The Bucket Tables were normalized for the Sum of Buckets and a two-sided student’s t-test was performed using a significance threshold of p<0.05 and a fold change >1.5. For extravascular tissue comparisons in fasting experiments, one ROI was used per biological replicate (n = 5 per group). For intravascular comparisons, one ROI was used per biological replicate (n = 3 per group), where the 3 replicates were selected based on which tissue cross-sections contained vascular regions of comparable size. For other comparisons, ROIs encapsulated the full tissue section. Heatmaps were constructed in OriginPro using a Ward clustering method and a Euclidean distance type for the dendrograms.

### Peak annotations

Spectral features were annotated by comparing experimental measurements to an in-house metabolite library within MetaboScape consisting of 114,008 compounds curated from the Human Metabolome Database (HMDB) version 4.0. The tolerance threshold was set to < 5ppm for tentative assignments. Using SCiLS Lab software, a total of ~1500 spectral peaks were processed from each MALDI MS image, and using a targeted approach, 68 compounds (S1 Table) were considered for analysis. On-tissue tandem MS measurements were performed to confirm peak annotations. The precursor mass was first selected by applying minimal collision energy to ensure the isolation window (3 mDa) was appropriate. Using collision-induced dissociation (CID), the energy was ramped from 5–50 eV and the collected spectrum was compared to spectra from the MSMS Metlin database. The Metabolomics Standards Initiative guidelines were used to classify the annotation confidence [26]. A level three characterization was based solely on accurate mass comparisons with reference databases. A level two characterization was based on both accurate mass and tandem MS measurements and compared with external databases for putative assignment. Using these guidelines, 37 metabolites were characterized as level three, and 31 were putatively assigned as level 2 (S1 Table).

### Dimensionality reduction and data visualization

Dimensionality reduction was used to enable interpretable visualization of the high dimensional spectra using Uniform Manifold Approximation and Projection (UMAP) [27]. The UMAP learns similarities of the mass spectra in the high-dimensional space and then projects it into a lower dimensional space of two dimensions, where similar spectra are projected close to each other, and dissimilar ones are projected further away. UMAP [27] was performed in an unsupervised manner and the reduced data was then colored based on the treatment (for heat inactivation experiments) or treatment, mouse ID, or metabolite of interest (for fasting experiments). The analysis was performed in R software (version 4.0.3) using the publicly available UMAP library and visualized using ggplot2 [27].

### Pathway enrichment analysis

Pathway analysis was performed using MetaboAnalyst 4.0 [28]. Metabolite features identified as significantly increased after fasting in Metaboscape were exported to MetaboAnalyst using the associated HMDB ID. The enrichment method used was a hypergeometric test and the topology analysis used was relative-betweenness centrality, with the KEGG reference library.

### Pathway visualization

Pathways of interest were constructed in PathVisio 3.3.0 [29] and imported into MetaboScape 6.0 (Bruker Daltonics) using the “Pathway Mapping” tool to visualize the relative changes in metabolite levels.

### Metabolite colocalization analysis

To determine colocalization of DHA and ARA, signal intensity plots for each metabolite were generated in Fiji (ImageJ 1.53c) (43). Ion images for DHA and ARA were exported from SCiLS Lab and converted to 16-bit in Fiji. Windows were synchronized and freehand lines were drawn between adjacent vessels. The intensity plots for each metabolite were then generated along this line, with metabolite signal intensity (gray value) as a function of distance between vessels (in pixels). Data were exported and visualized using GraphPad Prism 8.2.1 software (GraphPad Software).

### Metabolite extraction from tissue

Frozen tissues were maintained under dry ice vapor to remain frozen until extraction, and 10–20 mg was excised with a razor blade and samples were transferred to pre-chilled Eppendorf tubes. Extraction solution consisted of a pre-chilled (-20°C) solution of 2:2:1 HPLC-grade acetonitrile:methanol:water with 0.1 mol/L formic acid. Pre-chilled stainless-steel beads were added to Eppendorf tubes containing tissue samples, before extraction solution was added to achieve a concentration of 20 mg/mL before immediate lysis in a benchtop TissueLyser LT (Qiagen) operated at 50 Hz for 3 minutes. Next, 15% ammonium bicarbonate solution (filtered, room temperature) was added to achieve an 8% (v/v) solution and samples were lysed for another 3 minutes at 50 Hz. Samples were transferred to a benchtop shaker and vortexed at 4°C for 15 minutes. Beads were removed and samples were centrifuged at 16,000 × g at 4°C for 20 minutes. Clear supernatant was transferred to glass HPLC vials for immediate HPLC-MS analysis.

### HPLC-MS analysis

An iHILIC column (HILICON) was used with SII UPLC system (Thermo Fisher Scientific) coupled to a Q-Exactive HF-X orbitrap mass spectrometer (Thermo Fisher Scientific) operated with electrospray (ESI) ionization in negative ion mode at scan range *m/z* 75–1000 and a resolution of 60,000 at *m/z* 200. Buffer conditions used were: 20mM ammonium carbonate with 0.1% ammonium hydroxide in water (buffer A) and acetonitrile (buffer B). A flow rate of 0.150 mL/min was used with the following linear gradients: 0–20 min gradient from 80% to 20% B; 20–20.5 min gradient from 20% to 80% B; 20.5–28 min hold at 80% B; 28–30 min hold to waste at 80% B. Data were acquired using Xcalibur software (Thermo Fisher Scientific) and peak areas of metabolites were determined using TraceFinder 4.1 software (Thermo Fisher Scientific). Metabolites were identified by matching mass and retention time of features to commercial metabolite standards acquired previously on our instrument. Metabolite levels were normalized to tissue weight.

### Histology

Serial sections (10 μm) were fixed and stained using hematoxylin and eosin (H&E) immediately after sectioning and imaged using a bright field microscope (Zeiss Observer Z.1, Oberkochen, Germany) equipped with a Plan-APOCHROMAT lens and AxioCam MR3 camera, using a 20× or 40× magnification. High-resolution images of whole stained tissue sections were obtained using the stitching algorithm in Zeiss ZEN imaging software.

## Results

### Heat inactivation and desiccation treatments to reduce enzymatic breakdown of metabolites

Several experimental parameters needed to be assessed to faithfully recapitulate tissue metabolism *in situ* to visualize regions of metabolism in the liver. As major concerns are residual enzyme activity and non-enzymatic breakdown of labile metabolites, we evaluated whether enzyme inactivation through heat inactivation or desiccation treatments would stabilize tissue metabolites for MALDI MSI sample preparation. Heat inactivation was investigated as an alternative strategy to desiccation using kidney and brain tissues in addition to livers, as these organs have distinct anatomical features and metabolic compositions (S1A Fig). Control mouse tissues were resected and snap-frozen in liquid nitrogen before sectioning (Freeze, treatment_F_) and compared to fresh tissues subjected to heat inactivation to denature enzymes before freezing and sectioning (Heat-Freeze, treatment_HF_). The third group of tissues was snap-frozen to preserve the metabolic state immediately upon resection and then heat-treated to denature enzymes before re-freezing and sectioning (Freeze-Heat-Freeze, treatment_FHF_).

**Fig 1:**
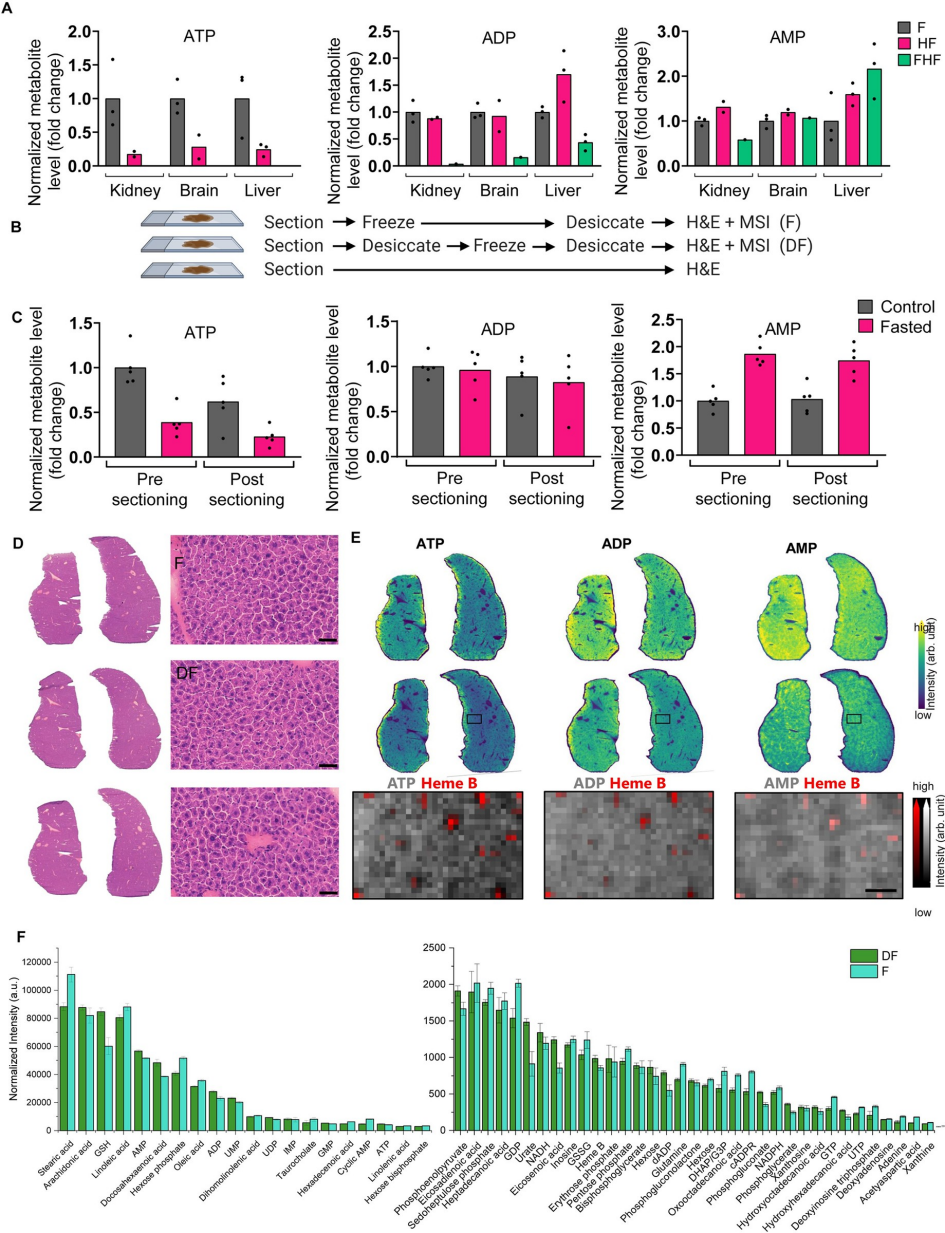
Evaluation of MALDI MSI sample preparation for small metabolites analysis. *(A)* Using a commercial heat stabilizing device, kidney, brain and liver tissues were subjected to freezing (treatment_F_), heat treatment followed by freezing (treatment_HF_, top) or treatment_FHF_.LC-MS relative quantification of total ATP, ADP, and AMP levels in the three tissues underwent varied heat treatments. *(B)* Schematic overview of treatments where serial tissue sections were either frozen at -80°C (treatment_F_), desiccated before freezing (treatment_DF_), or subjected to H&E staining directly after sectioning (H&E). (C) LC-MS relative quantification of total ATP, ADP, and AMP levels in liver tissues from control and fasted mice, before and after cryosectioning of serial sections for MALDI MSI analyses. *(D)* Histological images (20x magnification) of two mouse livers subjected to the treatments indicated in *(B)*. *(E)* Spatial mapping (30 μm pixel) of ATP, ADP, and AMP from the two liver tissue sections that underwent the treatments indicated in *(B)*. MSI ion images showing the relative distribution of ATP, ADP, and AMP individually or in relation to the vasculature indicated by heme B. *(F)* Bar graphs of annotated peak intensities using treatment_F_ and treatment_DF_.

Using a commercial device (Denator, Gothenburg, Sweden), conductive heating was applied to the whole tissue specimens using the predefined frozen or fresh settings. Histological evaluation of the three tissues showed that heat treatment disrupted tissue architecture, whereas this was preserved in frozen control tissues (S1B–S1D Fig). After the different heat treatments, LC-MS and MALDI-MSI were implemented to monitor the intensities and ion distributions of ATP, ADP, and AMP. Both the LC-MS data (Fig 1A) and MALDI MSI (S2 Fig) of the same tissue showed a relatively stable distribution upon treatment_HF_ as compared to control tissues, but a loss of overall ATP levels upon treatment_FHF_. As ATP use by enzymes will lead to increased levels of AMP and ADP, successful heat stabilization of enzymes should lead to stable levels of ATP, ADP, and AMP. This comparison showed that although heat stabilization of fresh tissue seemed to maintain the spatial distribution of adenosine phosphate metabolites seen in control tissues, intensity levels of ATP decreased.

MSI ion images were used to visualize the relative spatial distribution of metabolite levels based on peak intensities and revealed the overall loss of tissue morphology (S2C and S2D Fig). Since whole tissues were processed, the heating profiles needed to be optimized to provide uniform heating throughout the tissue; however, this was not possible due to the tissue’s thickness. Although regional clusters of metabolites in heat-treated brains were largely maintained, they could not be accurately mapped to anatomical brain regions due to the loss of tissue morphology. Together, these results indicate that the heat treatment applied to the whole tissue prior to sectioning led to disruption of tissue structure and compromised the integrity of anatomical regions. Further optimization of the heating profile for the denaturation of enzymes and the preservation of metabolites is needed for uniform stabilization that would be compatible with spatial metabolomics workflows.

Since enzymatic breakdown occurs at physiological conditions, it is critical that the storage and handling of tissues are managed with such consideration. In the MALDI MSI workflow, thaw-mounting a tissue section onto a slide can introduce higher temperatures within the tissue and introduce the breakdown of labile compounds. Thus, we focused on two potential anatomy-preserving methods to reduce enzymatic breakdown by comparing procedures of storing cryosectioned tissue on slides at -80°C and thawing them in a vacuum desiccator to minimize rehydration due to condensation (treatment_F_), as well as desiccation immediately after tissue sectioning before storage (treatment_DF_) (Fig 1B). To assess the stability of labile compounds from control and HFD liver tissues during the cryosectioning step, a thick tissue section was collected from the specimens at the beginning and at the end for comparison and processed for LC-MS. There was no indication of further ATP conversion to AMP over the time required to cryosection all specimens, allowing for the comparison of metabolite levels under different biological conditions (Fig 1C). To probe the tissue integrity based on treatment_F_ and treatment_DF_, serial liver sections were H&E stained for histological analysis immediately after sectioning to evaluate the effects of freezing and desiccation. Minimal gross tissue morphology differences were observed between the two treatments (Fig 1D). We used ATP stability as an indicator of postmortem enzymatic activity and labile metabolite stability, as it is used by many enzymes and is liable to degradation. Using both methods, the ATP, ADP, and AMP ion images showed comparable spatial distributions and metabolite levels (Fig 1E). When comparing the two methods, similar metabolite coverage was observed however, treatment_F_ showed higher signal intensity from several small compounds (Fig 1F), supporting the adoption of treatment_F_ for all subsequent sample preparation. Together, these results suggest that optimized MALDI MSI sample preparation and data acquisition workflow are needed to achieve broad coverage of small metabolites to generate reproducible spatial profiles of biologically relevant metabolic pathways.

### Distinct spatially-resolved metabolic signatures were observed in fed and fasted livers

**Fig 2:**
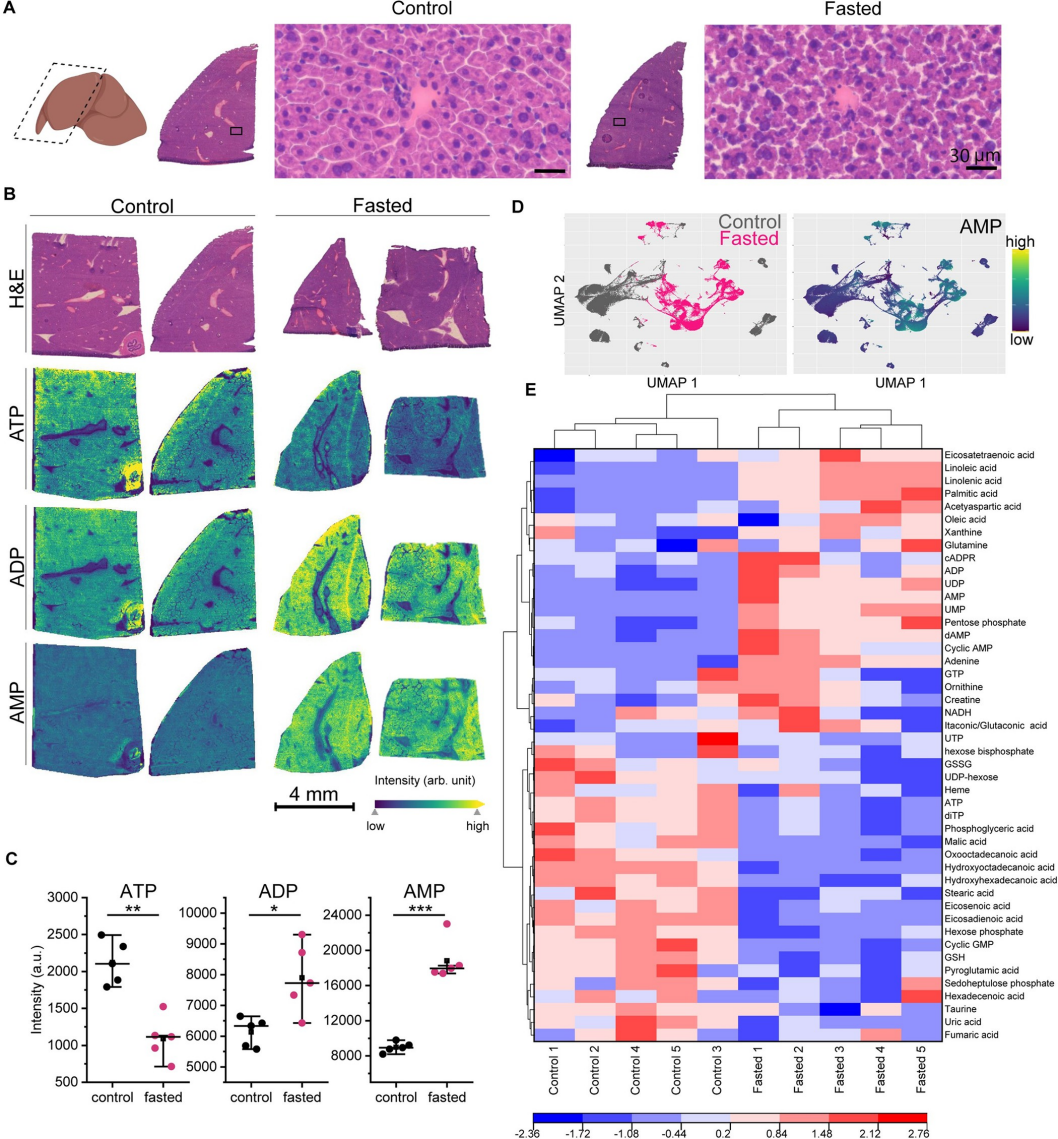
Spatially-resolved metabolic signatures in fed and fasted livers. *(A)* Histological images (40x magnification) of a representative liver section from *ad lib* fed mice and those subjected to overnight fasting; n = 5 per group. (*B*) H&E optical and MALDI MSI ion images (30 μm pixel) of representative serial tissue sections from control and fasted mice. MSI ion images show the relative distribution of ATP, ADP, and AMP. *(C)* Box-and-whisker plots of ATP, ADP, and AMP levels of the whole liver ROI from control and fasted mice. *P < 0.05, **P <0.01, ***P < 0.001, ****P < 0.0001 (Student’s t-test) *(D)* UMAP non-linear dimensionality reduction of MALDI MSI data from liver tissues from *ad lib* fed mice or those subjected to an overnight fast showed distinct group clustering. The AMP ion intensity plotted onto the UMAP revealed higher abundance in fasted conditions. *(E)* Heatmap of 46 metabolite levels plotted according to fasted and control states, indicating a metabolic response based on treatment.

Regions of metabolism were investigated in the liver in response to fasting by generating spatially-resolved metabolic profiles. Livers from fasted mice showed marked histological differences in hepatocyte shape due to the expected depletion of glycogen (Fig 2A). Using MSI, we observed that fasting led to a decrease in liver ATP content with a concomitant increase in AMP, indicative of cellular nutrient stress (Figs 2B, S3C and S3D). Furthermore, by plotting the ion intensities of these purines for both control and fasted livers (n = 5), we observed that ATP was significantly elevated in control, while ADP and AMP were significantly higher in abundance in the fasted tissues (Fig 2C). Group clustering of control and fasted spectral ions were observed based on a pixel point UMAP analysis, in which plotting the AMP signal intensity over the UMAP map revealed high correlation with the fasted group (Fig 2D). A comparison of mean spectra revealed marked differences in several other metabolite signal intensities between control and fasted mice (S3A and S3B Fig). Thus taking a targeted approach, a heatmap was constructed for metabolites related to the TCA, purine, glycolysis, fatty acid, and glutathione pathways (Fig 2E and S1 Table), revealing a higher abundance of unsaturated fatty acids and purine compounds associated with fasting, while several TCA metabolites were higher in abundance in the control livers.

### Visualization of fasted liver metabolism shows disruption of metabolism and fuel switching

**Fig 3:**
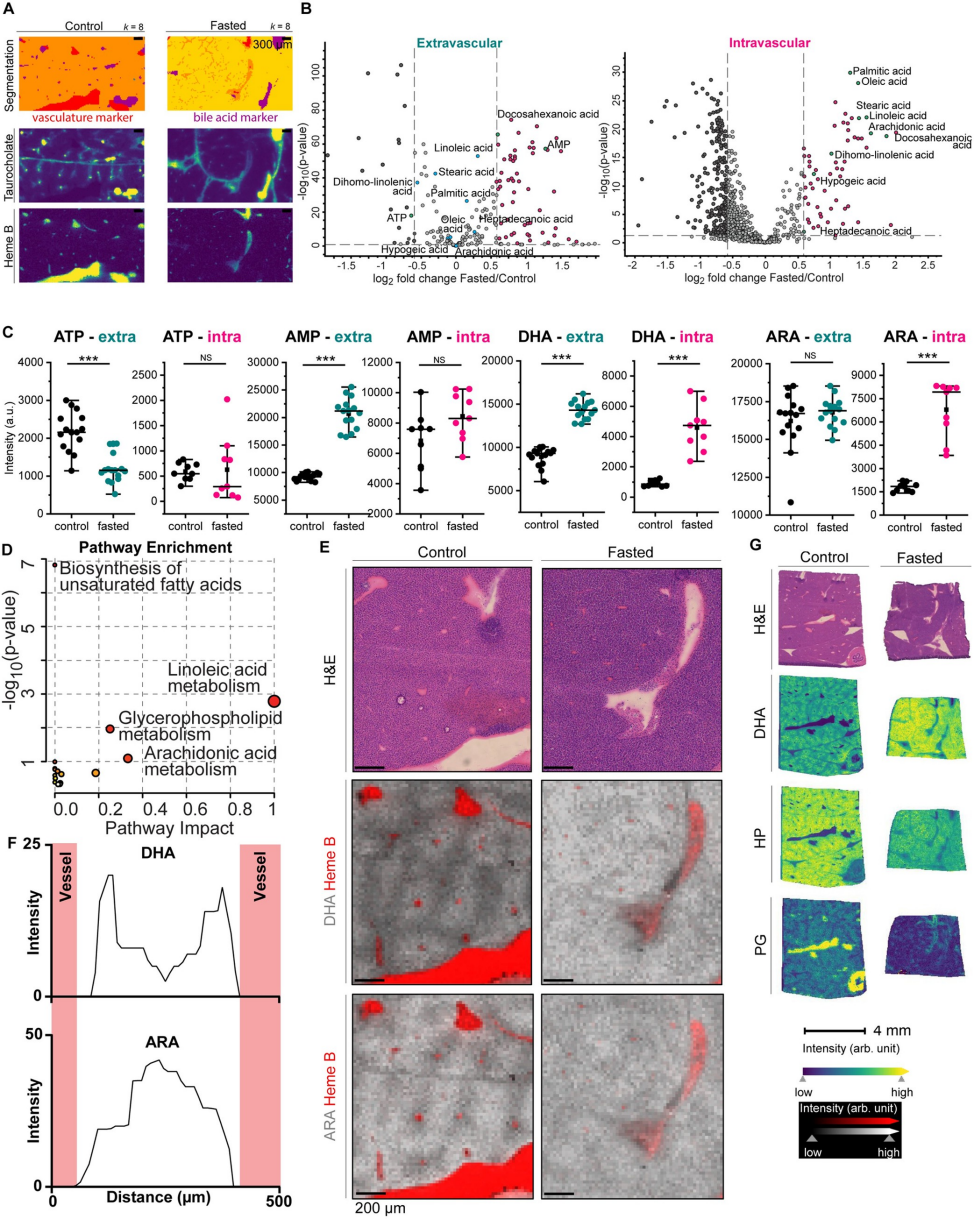
Liver metabolism and fuel switching. *(A*) Segmentation map of the MALDI MSI data based on bisecting k-means clustering (*k* = 8), where each cluster is represented as an individual color, and MALDI MSI ion images of heme B as a marker of the vasculature corresponding to the red segment and taurocholate as a marker of the bile acids corresponding to the purple segment. Intravascular regions were defined based on the intensity of heme B. *(B)* Volcano scatterplot displaying log 2 metabolite intensity ratios vs. significance value in fasted compared to control mouse liver extravascular (left) and intravascular tissue (right). Every point represents a unique metabolite; dark grey circles indicate metabolites depleted after fasting and magenta circles indicate metabolites enriched after fasting, that showed a fold change >1.5 between treatments and reached statistical significance (p-value <0.05). Highlighted green circles are statistically significantly changed metabolites indicating cellular energy status (AMP/ATP) and fatty acids with their corresponding names. Corresponding metabolites that were not statistically significantly changed are highlighted in blue. *(C)* MALDI MSI relative quantification of selected metabolites in the extravascular versus intravascular tissue regions. *P < 0.05, **P <0.01, ***P < 0.001, ****P < 0.0001 (Student’s t-test) *(D)* Pathway enrichment scatterplot displaying pathway impact scores vs. significance value in fasted compared to control mouse vasculature. Increased circle size indicates pathway coverage of the identified metabolites in the dataset. Pathways identified as enriched are displayed by name. *(E)* H&E and MALDI MSI ion images (30 μm pixel) of serial tissue sections from representative control and fasted mouse livers. MSI ion images show the relative distribution of DHA and ARA in relation to heme B in red, with indicated intensity scale. *(F)* Quantification of metabolite spatial distribution for DHA and ARA from blood vessel to an adjacent blood vessel, where the metabolite intensity is shown as a function of distance between two vessels. The vasculature position is indicated in red. *(G)* H&E and MALDI MSI ion images (30 μm pixel) of tissue serial sections from a representative control and fasted mouse liver. MSI ion images show relative distribution of DHA, HP, and PG, with indicated intensity scale.

**Fig 4:**
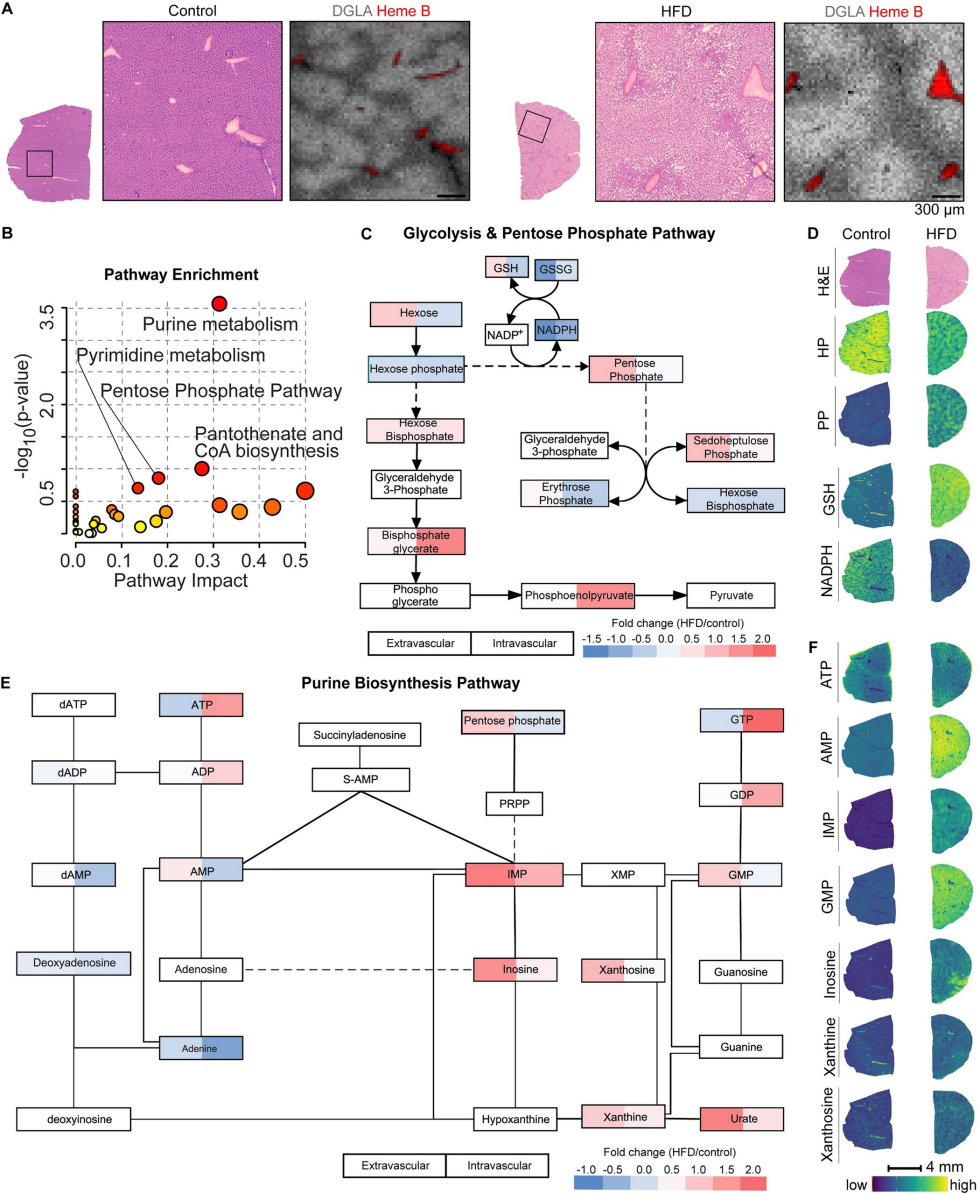
Fatty livers demonstrate oxidative stress and increased purine metabolites in response to prolonged nutrient excess. *(A*) Histological images of a representative liver section from *ad lib* fed mice on a control or high-fat diet for 4.5 months (n = 5 per group, 2 independent experiments) with the corresponding ion image of the fatty acid dihomo-linolenic acid (DGLA). *(B)* Pathway enrichment scatterplot displaying pathway impact scores vs. significance value in HFD compared to control mouse liver tissues. Increased circle size indicates pathway coverage of the identified metabolites in the dataset. Pathways identified as enriched are displayed by name. *(C)* Schematic overview of the connected metabolic pathways of glycolysis and the pentose phosphate pathway with corresponding relative fold change intensities of HFD compared to control mice, with indicated intensity scale. *(D)* H&E and MALDI MSI ion images from glycolysis pathway and PPP of tissue serial sections from a representative control and HFD mouse liver. MSI ion images(30 μm pixel) show relative distribution of the indicated metabolites, with indicated intensity scale. *(E)* Schematic overview of purine metabolism with corresponding relative fold change intensities of HFD compared to control mice, with indicated intensity scale.*(F)* MALDI MSI ion images of tissue serial sections from a representative control and HFD mouse liver showing relative distribution of the indicated metabolites, with indicated intensity scale.

To visualize these differences in an unbiased manner, we constructed a segmentation map (Fig 3A). This visualization showed distinct metabolic clusters within different anatomical regions of the liver and between control and fasted mice, while all biological replicates within each group clustered together (S4 Fig). Metabolite clusters were observed for the vasculature, hepatocytes, and bile acids. These clusters corresponded to co-registered ion images of heme B, a cofactor of hemoglobin that is enriched within the vasculature, and taurocholate, the most abundant bile acid (Fig 4A) [30,31].

The liver acts as a metabolic rheostat to maintain whole-body energy homeostasis in times of nutrient stress and excess. As MSI adds a spatial dimension to metabolomic analyses, we dissected the metabolic compartmentalization in the fasting liver. We identified metabolic differences using the unbiased UMAP approach, which showed separation of data clusters from fasted compared to control livers (Figs 2D and S4). Additional clusters were observed within treatment groups, visualized by coloring UMAP distributions per individual mouse (S4C and S4D Fig). The distribution of heme over the UMAP graphs indicated that these clusters could represent distinct anatomical regions within tissues (S4C Fig). To explore differences between liver and systemic metabolism, we extracted metabolite spectra from MSI data on a pixel-by-pixel basis. We used a segmentation map (Figs 3A, S5A and S5B) to select regions-of-interest enriched for hepatocytes (extravascular tissue) or heme B (intravascular tissue, circulating metabolites). Examining a volcano plot revealed ATP to be significantly decreased and AMP significantly increased in extravascular tissue upon fasting in accordance with our previous observations (Fig 3B, left). We also observed an increase in the fatty acid docosahexaenoic acid (DHA). This was recapitulated in the UMAP distributions, where AMP and DHA were more abundant in fasted mice (S4D Fig). The metabolite profiles from intravascular regions did not show differences in adenosine phosphate metabolites, but several fatty acids were significantly enriched in the circulation upon fasting (Fig 3B, right and Fig 3C) whereas they were not significantly changed within extravascular regions of the tissue (S5C Fig). It is well-understood that the adipose tissue releases fatty acids for oxidation by the liver to yield ketone bodies that can fuel distant organs, which is corroborated by these results and indicates that spatially-resolved metabolomics can inform on metabolic compartmentalization within tissues.

Indeed, pathway analysis of the intravascular regions showed that several lipid metabolic pathways were enriched (Fig 3D and S2 Table). Interestingly, comparing the spatial distribution of fatty acids showed that the abundance of DHA and ARA follow a specific and compartmentalization pattern in fed livers (Figs 3E and S5B). DHA is a 22-carbon polyunsaturated omega-3 fatty acid (22:6), whereas arachidonic acid (ARA) is a 20-carbon polyunsaturated omega-6 fatty acid (20:4). Both can be synthesized from alpha-linolenic acid, which in turn is produced from the essential fatty acid linoleic acid. These fatty acids can also be released from complex lipids through lipolysis. Plotting a line profile of the metabolite intensity as a function of the distance between blood vessels confirmed that DHA is enriched in proximity to the vasculature while ARA displayed the opposite enrichment pattern (Figs 3F and S5D). Upon fasting, this distinct spatial compartmentalization within the extravascular regions is lost. In contrast to the increase in DHA within liver cells, the levels of glycolytic intermediates decreased within liver tissue, indicating a fuel switch upon fasting that decreases liver glucose use in favor of lipid metabolism (Fig 3G). Together, these results indicate that spatially dissecting metabolite profiles can yield new insights into metabolic compartmentalization within tissues and between the local tissue environment and the circulation.

### Fatty livers show a metabolic signature indicative of oxidative stress in response to prolonged nutrient excess

We also investigated how the liver’s response to nutrient excess might impact region specific metabolism by subjecting mice to a high-fat diet (HFD). Livers of HFD mice showed marked histological differences, with hypertrophy and accumulation of lipid droplets that displayed in unique patterns where lipid droplets were deposited away from the vasculature (Fig 4A). In human, it has been established that macrovesicular steatosis, where hepatocytes become displaced by lipid droplets, is associated with advanced fatty liver disease, inflammation, fibrosis, and poor clinical outcomes [32,33]. We evaluated the changes in metabolite levels, and subsequent pathway analysis showed that several metabolic pathways were significantly enriched upon HFD feeding, including the pentose phosphate pathway and purine metabolism (Fig 4B). Cells increase PPP activity in response to oxidative stress to generate NADPH, a reducing factor that is essential to maintain reduced pools of glutathione, the main antioxidant in cells, and antioxidant enzymes that help maintain cellular redox balance (Fig 4C). That HFD livers experience increased redox stress is suggested by the observed increase in glutathione in the extravascular tissue regions (GSH; Fig 4C and 4D). Interestingly, although the PPP intermediates pentose phosphate (PP) and sedoheptulose phosphate (SP) are increased in extravascular tissue regions of fatty livers and not changed in the intravascular regions, levels of NADPH are decreased in both the intravascular and extravascular tissue regions (Fig 4C). This finding suggests that despite the cellular reprogramming towards an antioxidant response that occurs in fatty livers, cells have lower NADPH levels.

Pathway enrichment analysis showed that in addition to the PPP, purine metabolism was significantly enriched in fatty livers (Fig 4B and S3 Table). Purines are essential for supplying the building blocks for nucleotides, thereby DNA/RNA synthesis, and nucleotide cofactors such as NAD and the major energy carriers in cells (Figs 4E and S6). Increases in redox stress are known to increase DNA damage and might trigger purine metabolism to aid DNA repair, whereas the disruption of cellular energy status may converge upon the purine and pyrimidine pathways due to their important roles as cellular energy carriers to maintain cellular homeostasis. Together, these results suggest that spatially dissecting metabolite profiles and multiplexing tissue anatomical information with metabolic characterization can promote our understanding of metabolic compartmentalization in physiology and pathology.

## Discussion

Metabolic heterogeneity within tissues and metabolic crosstalk between cells are essential contributors to functional specialization in multicellular organisms. This emphasizes the need to introduce spatiality into metabolomic analyses to better understand the role of metabolic heterogeneity in physiology and disease. MALDI MSI has been used to study protein, drug and metabolite distribution in tissues from model organisms and humans to yield new biological insights. Spatially mapping endogenous metabolites can be applied to delineate metabolic properties of distinct anatomical structures [34], inform on their biological functions [35], identify abnormal or pathological regions within tissues [36], and their metabolic properties [37], and aid in surgical decision-making [38]. Advances in instrumentation and application have produced increased molecular complexity and spatial resolution analyses leading to new insights into metabolic function and heterogeneity at the single-cell scale [19,39]. With increasing sensitivity and specificity in ion detection and annotation, MSI is now emerging as a tool for spatially-resolved, metabolome-scale analyses that advance our understanding of cellular and organismal biology [39]. Maintaining metabolic fidelity of the tissue during sample processing is essential to yielding meaningful analyses, especially in comparison with chromatography-based mass spectrometry approaches where metabolomes are stabilized by quenching steps and samples are maintained at low temperatures until analysis while several sample preparation steps for MALDI MSI occur at ambient conditions. Here, we demonstrate an approach to prepare tissue samples for MSI that minimizes conversion or breakdown of labile metabolites while broadening the range of small metabolites detected to more broadly cover metabolic pathways and yield new insights into tissue metabolism.

Liver zonation is well-understood on the transcript level [2,3,5,6,9,40,41], but has not been comprehensively visualized on the metabolite level. An important advance of profiling metabolic heterogeneity on the metabolite rather than transcript level is that an immediate snapshot of metabolism can be captured instead of indirect measures provided by enzyme transcripts or protein levels. Direct metabolite profiling is enabled by the fact that MALDI MSI requires minimal sample handling, and dissociation of distinct cell types is not necessary. We were able to validate and visualize metabolic compartmentalization in liver tissues in distinct nutrient stress and excess conditions. We observed distinct metabolic profiles within zones and between tissue compartments, which may be obscured in extraction-based metabolomic analyses as the hepatocyte fraction contributes most of the mass and metabolic content of the liver. By analyzing metabolite spectra from distinct extra- and intravascular regions, we observed specific metabolic profiles consistent with the known metabolic function of each compartment. We observed a strong enrichment of fatty acids in blood vessels, consistent with the liver’s function of converting fatty acids released from the adipose tissue to generate alternative fuels for distant organs. In addition to compartmentalization between the liver organ environment and the circulation, we also observed distinct patterns of metabolite abundance within the tissue microenvironment, with hepatocytes showing enrichment of specific fatty acids based on their proximity to the vasculature. This distinct pattern was highly organized and reproducible between biological replicates in nutrient-replete conditions but vanished when facing nutrient stress after fasting. This suggests that prolonged nutrient stress induces metabolic adaptations that overrule the functional compartmentalization of hepatocytes seen under nutrient-replete conditions. In prolonged nutrient excess conditions induced by a high-fat diet, lipid droplets accumulate in the liver, forming distinct lipid depots throughout the tissue.

In contrast to fasting conditions, where glycolytic metabolites were low, fatty livers displayed higher levels of glycolytic and PPP metabolites together with a marked increase in GSH levels, indicating oxidative stress. Additionally, we observed an increase in purine metabolism, which may produce nucleotides needed to repair DNA damage, generate essential energy carriers, or provide precursors for metabolic cofactors such as NAD, which can all become disturbed by cellular redox stress. These results indicate that although the lipid content of the liver increases upon HFD feeding, the lipid droplets act as an overflow depot rather than being effectively metabolized by the liver to dissipate excess energy. Adding a temporal component to our spatial metabolomic analyses and multiplexing with orthogonal modes of single-cell tissue imaging analyses [42,43] may help further elucidate which regulatory nodes govern the observed fuel switching in fasting and fatty liver. Taken together, our described workflow enables the detection of endogenous metabolites and achieves a broad coverage of the tissue metabolome that can be applied to characterize and interrogate metabolic heterogeneity in physiology and pathology. In this study, the right lobe of a mouse liver was used, however, due to the large size and complex heterogeneity nature of the liver, scaling this to human livers would require histological selection of tissue specimens to allow for image comparison of different health and disease states.

## Conclusions

Cellular metabolism is spatiotemporally heterogeneous, yet leading metabolomics approaches do not preserve spatial information. We present a MALDI MSI approach to map metabolic heterogeneity in the liver in nutrient replete, stress, and excess conditions. Our data validate and extend what is known about liver metabolic compartmentalization and visualize this at high resolution with broad coverage of key pathways in central energy metabolism. The label-free molecular imaging approach demonstrated here can be applied broadly to study metabolism in tissues and reveal new insights into metabolic heterogeneity *in vivo* to better understand the role of metabolism in physiology and pathology.

## Supporting information

S1 Fig Heat treatment disrupts tissue integrity. (PDF)

S2 Fig Heat treatment causes interconversion and breakdown of adenosine phosphate metabolites. (PDF)

S3 Fig Comprehensive spatial metabolic imaging reveals distinct spatially-resolved metabolic signatures in fed and fasted livers. (PDF)

S4 Fig Nutrient stress disrupts liver metabolic zonation and causes fuel 48 switching to maintain whole-body metabolic homeostasis. (PDF)

S5 Fig Nutrient stress disrupts liver metabolic zonation and causes fuel 62 switching to maintain whole-body metabolic homeostasis. (PDF)

S6 Fig Fatty livers face oxidative stress and increase purine metabolism 78 in response to prolonged nutrient excess. (PDF)

S1 Table Metabolite annotations. (PDF)

S2 Table Pathway enrichment of liver metabolism and fuel switching. (PDF)

S3 Table Pathway enrichment in fatty liver disease. (PDF)
