# Supplementary material for: Spatially resolved characterization of tissue metabolic compartments in fasted and high-fat diet livers
Source: PLoS One. 2022 Sep 6;17(9):e0261803. doi: 10.1371/journal.pone.0261803 (PMC9447892; doi:10.1371/journal.pone.0261803)
Supplement: S1 Fig — (PDF) [file pone.0261803.s001.pdf]

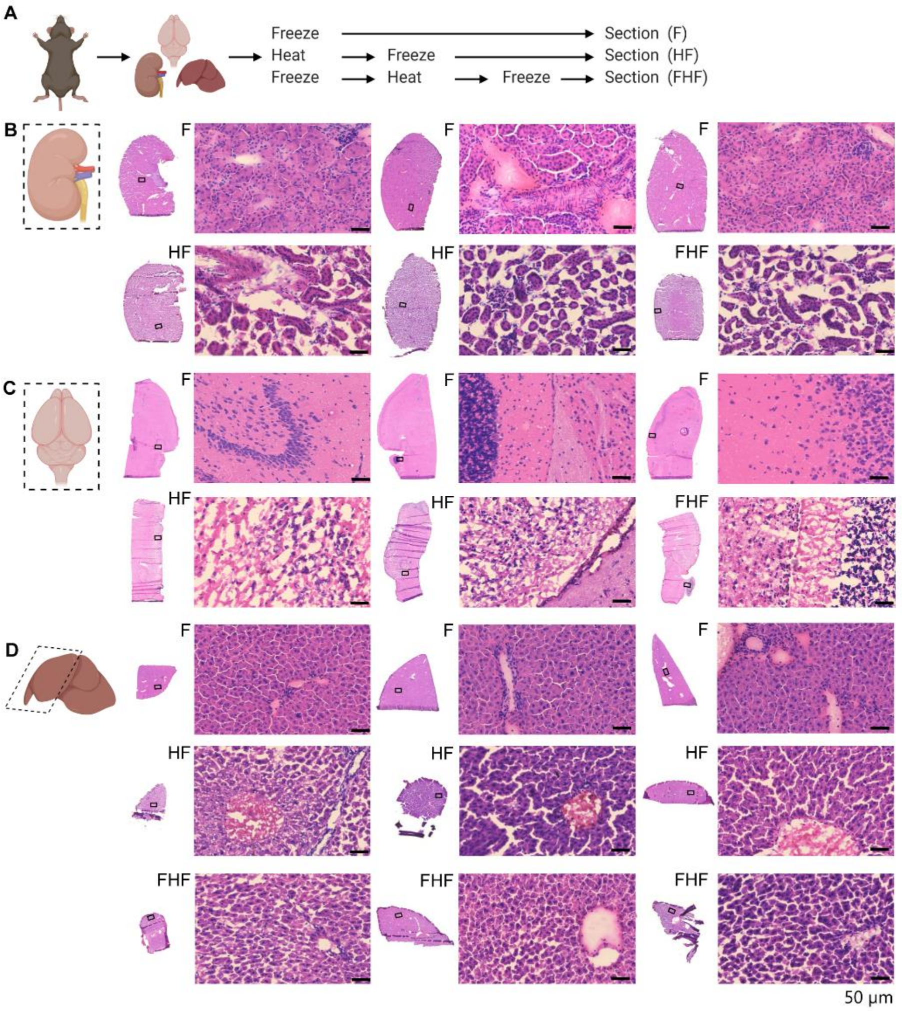

E

Mouse	Organ	Section	Treatment	Heat inactivation (seconds)	H&E and MSI images
	Kidney	Left	F	-	Supplementary Figure 1B
		Right	HF	37	Supplementary Figure 2C
**1**	Brain	Left	HF	28	Supplementary Figure 2C
		Right	F	-	Supplementary Figure 1C
	Liver	Middle lobe	F	-	Supplementary Figure 2C
		Left lobe	HF	37	Supplementary Figure 1D
		Right lobe	FHF	44	Supplementary Figure 1D
	Kidney	Left	F	-	Supplementary Figure 1B
		Right	FHF	40	Supplementary Figure 2C
**2**	Brain	Left	FHF	56*	Supplementary Figure 2C
		Right	F	-	Supplementary Figure 1C
	Liver	Middle lobe	FHF	37	Supplementary Figure 2C
		Left lobe	F	-	Supplementary Figure 1D
		Right lobe	HF	37	Supplementary Figure 1D
	Kidney	Left	HF	39	Supplementary Figure 1B
		Right	F	-	Supplementary Figure 2C
**3**	Brain	Left	F	-	Supplementary Figure 2C
		Right	HF	39	Supplementary Figure 1C
	Liver	Middle lobe	HF	39	Supplementary Figure 2C
		Left lobe	FHF	30	Supplementary Figure 1D
		Right lobe	F	-	Supplementary Figure 1D

Supplementary Figure 1. Heat treatment disrupts tissue integrity. *(A)* Schematic overview of tissue treatments where resected organs were either snap-frozen in liquid nitrogen ($treatment_F$), heat-treated and then frozen ($treatment_{HF}$), or frozen before heat treatment and re-frozen ($treatment_{FHF}$). Histological (H&E) images at 20x magnification of coronal tissue sections of kidney *(B)*, brain *(C)*, and liver *(D)* that were subjected to the varied freezing and heat treatments. Regions of interest are shown in insets to evaluate tissue integrity. *(E)* Overview of heat treatment duration per tissue type and replicates. Asterisk indicates one instance where the Maintainor® tissue cards used with the Stabilizor™ heat inactivation system did not seal fully, which may have led to prolonged heat exposure.
