# Supplementary material for: Spatially resolved characterization of tissue metabolic compartments in fasted and high-fat diet livers
Source: PLoS One. 2022 Sep 6;17(9):e0261803. doi: 10.1371/journal.pone.0261803 (PMC9447892; doi:10.1371/journal.pone.0261803)
Supplement: S2 Fig — (PDF) [file pone.0261803.s002.pdf]

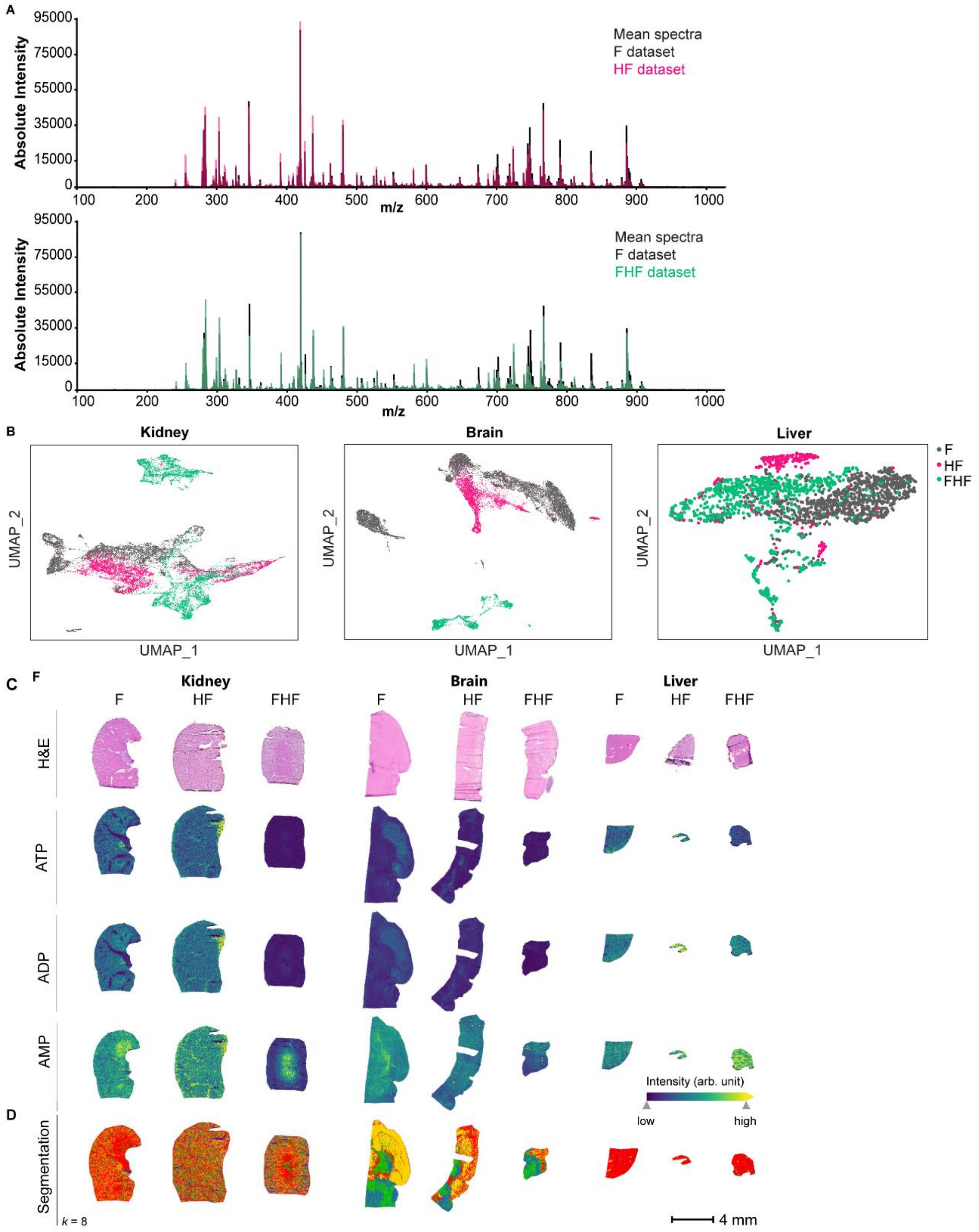
A
Absolute Intensity
Mean spectra
F dataset
HF dataset
m/z
Mean spectra
F dataset
FHF dataset
B
Kidney
Brain
Liver
UMAP_1
UMAP_2
F
HF
FHF
C
F
Kidney
Brain
Liver
HF
FHF
H&E
ATP
ADP
AMP
Intensity (arb. unit)
low
high
D
Segmentation
k = 8
4 mm

26

Supplementary Figure 2. Heat treatment causes interconversion and breakdown of adenosine phosphate metabolites. *(A)* MALDI MSI mean spectra overlays comparing data from tissues subjected to freezing (treatment$_{F}$) and heat treatment followed by freezing (treatment$_{HF}$, top) or treatment$_{FHF}$ (bottom). *(B)* UMAP non-linear dimensionality reduction of MALDI MSI data for kidney (left), brain (middle) and liver (right), showing distinct data clusters based on treatment. *(C)* H&E optical and molecular ion images of kidney (left), brain (middle), and liver (right) serial sections that were subjected to the varied freezing and heat treatments. MSI ion images showing relative distribution of ATP, ADP, and AMP. *(D)* Segmentation map of the MALDI MSI data based on bisecting k-means clustering (k = 8), where each cluster is represented as an individual color.
