# Supplementary material for: Spatially resolved characterization of tissue metabolic compartments in fasted and high-fat diet livers
Source: PLoS One. 2022 Sep 6;17(9):e0261803. doi: 10.1371/journal.pone.0261803 (PMC9447892; doi:10.1371/journal.pone.0261803)
Supplement: S3 Fig — (PDF) [file pone.0261803.s003.pdf]

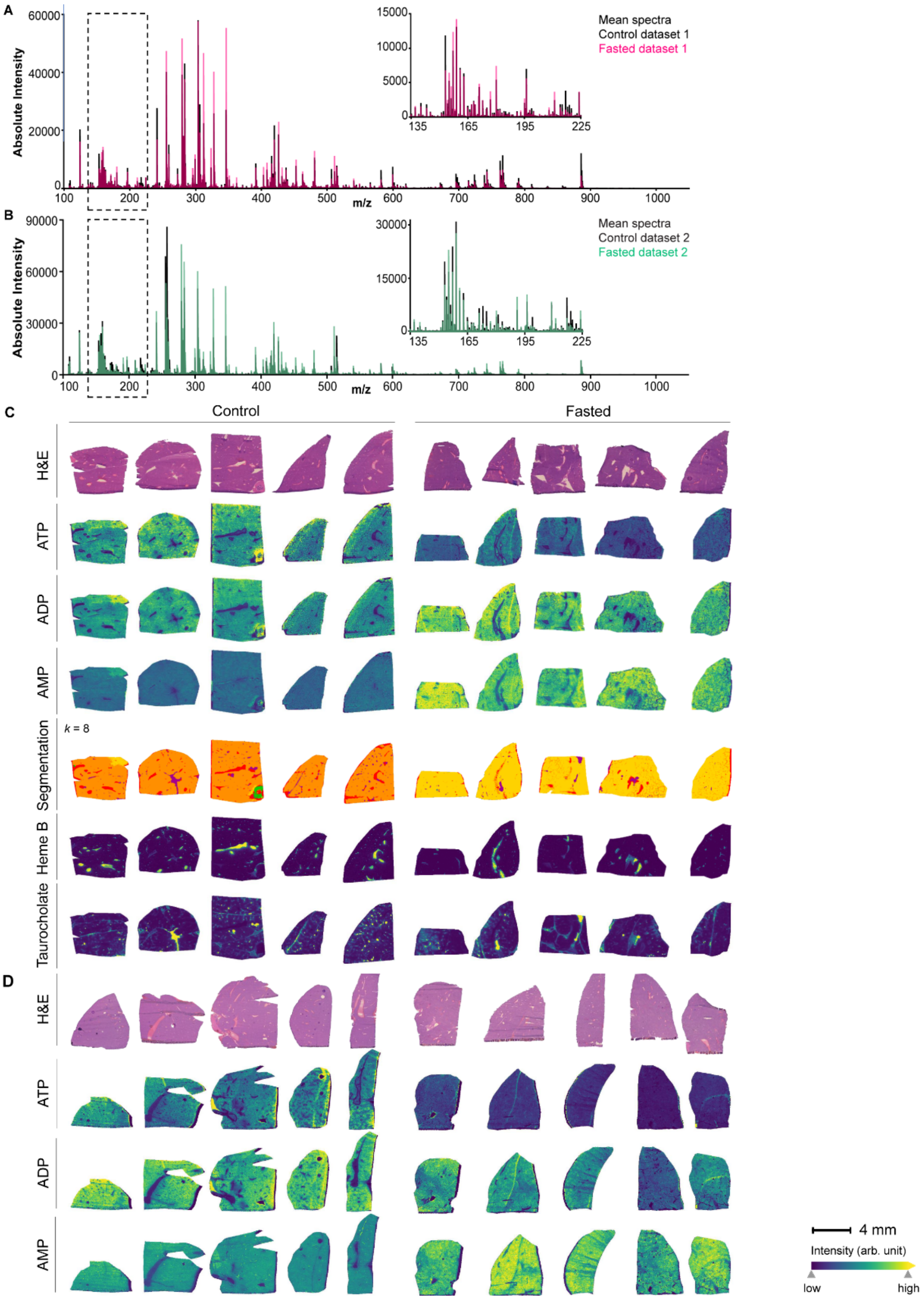
A
Absolute Intensity
m/z
Mean spectra
Control dataset 1
Fasted dataset 1
B
Absolute Intensity
m/z
Mean spectra
Control dataset 2
Fasted dataset 2
C
Control
Fasted
H&E
ATP
ADP
AMP
Segmentation
k = 8
Heme B
Taurocholate
D
H&E
ATP
ADP
AMP
4 mm
Intensity (arb. unit)
low
high

36

Supplementary Figure 3. Comprehensive spatial metabolic imaging reveals distinct spatially-resolved metabolic signatures in fed and fasted livers. *(A-B)* MALDI MSI mean spectra overlays comparing data from liver tissues from *ad lib* fed mice or those subjected to an overnight fast (n=5 per group) for two independent experiments (denoted as dataset 1 and 2). Inset highlights the small metabolite range between *m/z* 135-225 for the two treatments. *(C)* H&E and MALDI MSI ion images of tissue serial sections from control and fasted mice. MSI ion images show relative distribution of ATP, ADP, AMP, heme B as a marker of the vasculature, and taurocholate as a marker of the bile acids, in additon to the representative images shown in Figure 2. *(D)* H&E and MALDI MSI ion images of tissue serial sections from the second independent cohort of control and fasted mice. MSI ion images show relative distribution of ATP, ADP, and AMP.
