# Supplementary material for: Spatially resolved characterization of tissue metabolic compartments in fasted and high-fat diet livers
Source: PLoS One. 2022 Sep 6;17(9):e0261803. doi: 10.1371/journal.pone.0261803 (PMC9447892; doi:10.1371/journal.pone.0261803)
Supplement: S4 Fig — (PDF) [file pone.0261803.s004.pdf]

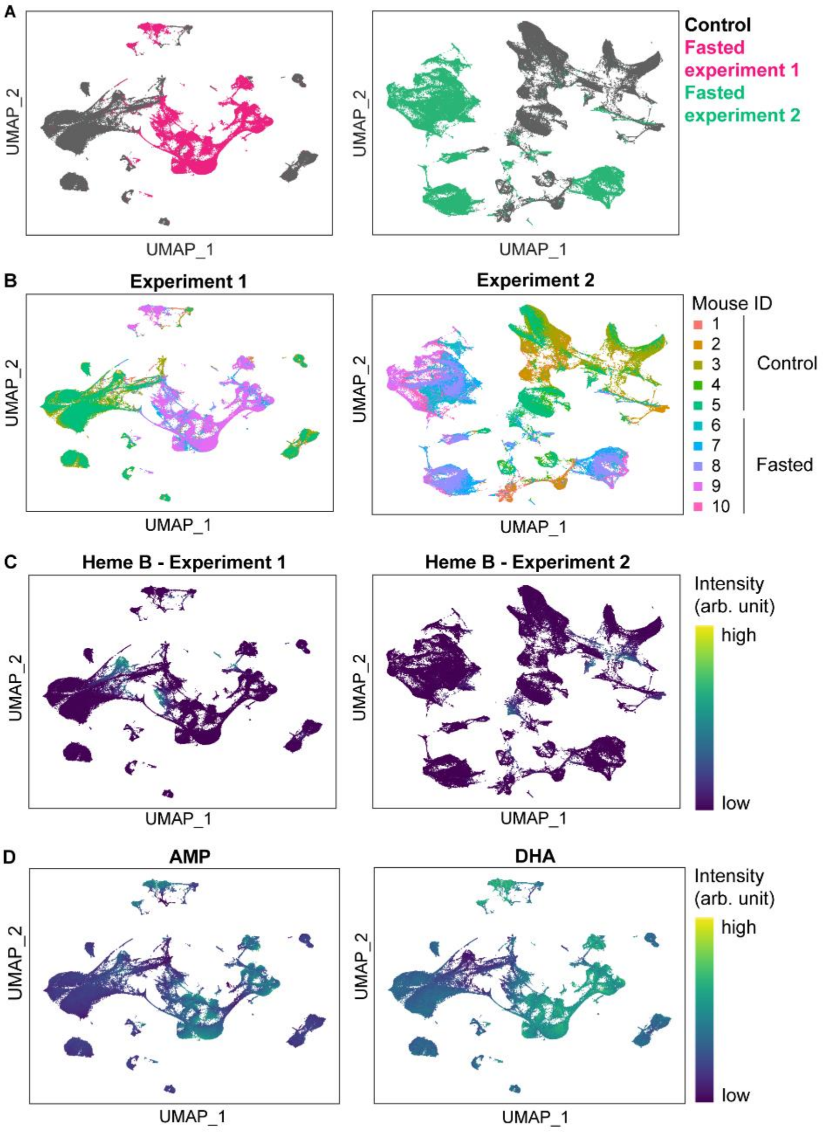
A
UMAP_2
UMAP_1
Control
Fasted
experiment 1
Fasted
experiment 2
B
Experiment 1
Experiment 2
Mouse ID
1
2
3
4
5
Control
6
7
8
9
10
Fasted
C
Heme B - Experiment 1
Heme B - Experiment 2
Intensity
(arb. unit)
high
low
D
AMP
DHA

47

Supplementary Figure 4. Nutrient stress disrupts liver metabolic zonation and causes fuel switching to maintain whole-body metabolic homeostasis. *(A)* UMAP non-linear dimensionality reduction of MALDI MSI data from liver tissues from *ad lib* fed mice or those subjected to an overnight fast for two independent experiments (denoted as experiment 1 and 2, n=5 per group), showing distinct data clusters based on treatment. *(B)* UMAP non-linear dimensionality reduction of MALDI MSI data from liver tissues from *ad lib* fed mice or those subjected to an overnight fast for two independent experiments (denoted as dataset 1 and 2, n=5 per group), where datapoints are labelled per individual mouse (n=5 per group). *(C)* UMAP non-linear dimensionality reduction of MALDI MSI data where datapoints are labelled based on the distribution of heme B as a marker for the vasculature with indicated intensity scale for two independent experiments. *(D)* UMAP non-linear dimensionality reduction where datapoints are labelled based on the distribution of metabolites AMP (left) and docosahexaenoic acid (DHA, right) with indicated intensity scale for experiment 1.
