# Supplementary material for: Spatially resolved characterization of tissue metabolic compartments in fasted and high-fat diet livers
Source: PLoS One. 2022 Sep 6;17(9):e0261803. doi: 10.1371/journal.pone.0261803 (PMC9447892; doi:10.1371/journal.pone.0261803)
Supplement: S5 Fig — (PDF) [file pone.0261803.s005.pdf]

61

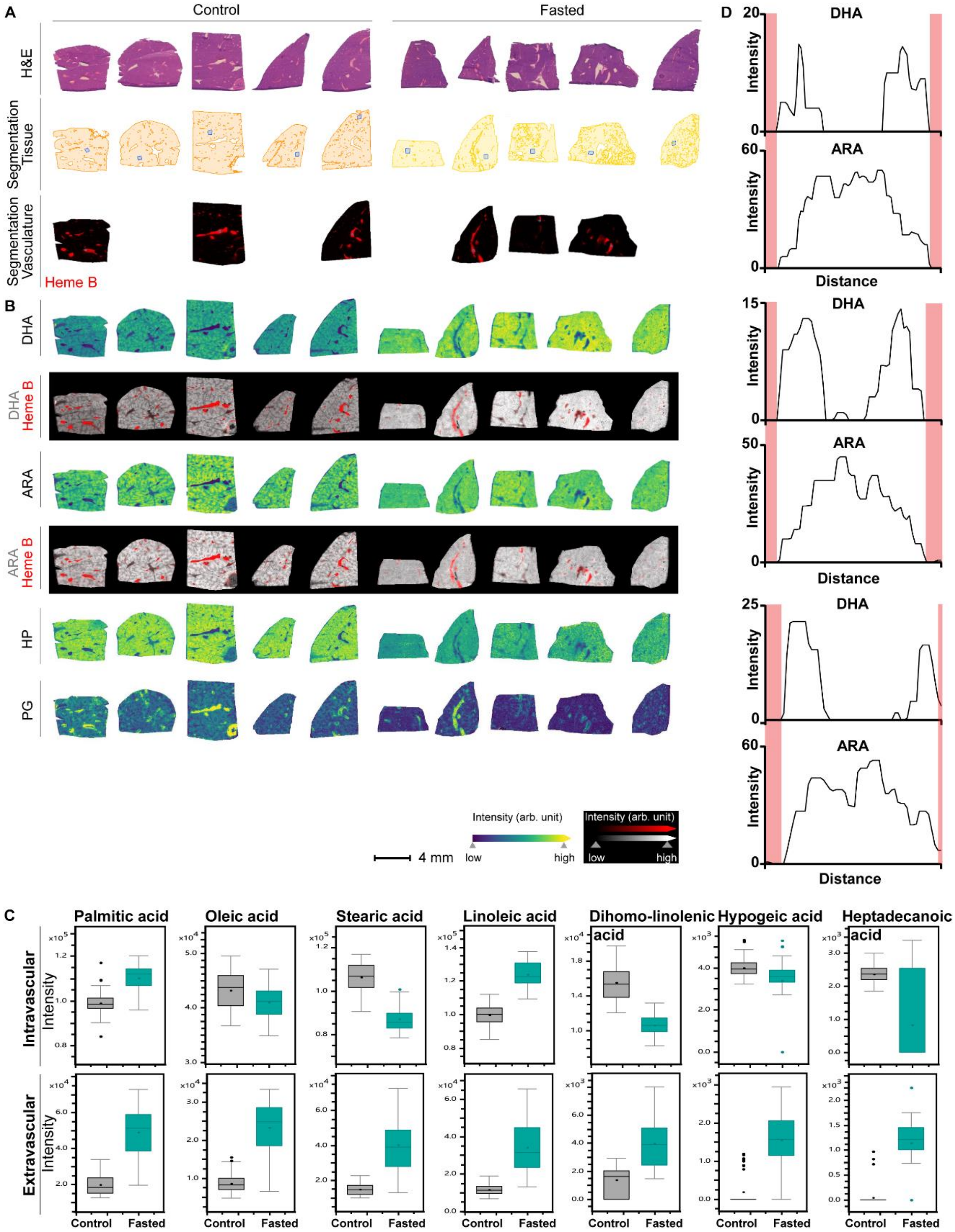

A
Control
Fasted
H&E
Segmentation Tissue
Segmentation Vasculature
Heme B
B
DHA
DHA Heme B
ARA
ARA Heme B
HP
PG
Intensity (arb. unit)
low
high
4 mm
C
Palmitic acid
Oleic acid
Stearic acid
Linoleic acid
Dihomo-linolenic acid
Hypogeic acid
Heptadecanoic acid
Intravascular
Extravascular
Intensity
Control
Fasted
D
DHA
ARA
Intensity
Distance

Supplementary Figure 5. Nutrient stress disrupts liver metabolic zonation and causes fuel switching to maintain whole-body metabolic homeostasis. *(A)* H&E, segmentation, and MALDI MSI ion images of tissue serial sections from control and fasted mice indicating how tissue and vasculature regions were defined for spatial metabolic analyses. Hepatocyte-enriched regions (denoted as Tissue) were identified using the segmentation map of the MALDI MSI data based on bisecting k-means clustering (k = 8), with control and fasted tissues represented as dark and light orange, respectively. Vascular regions were defined based on intensity of heme B (denoted as Vasculature). ROIs depicted in blue indicate where metabolite spectra were extracted for further spatial analysis. *(B)* MALDI MSI ion images of tissue serial sections from control and fasted mice showing the relative distribution of DHA and ARA individually and in relation to heme B, HP, and PG, in addition to the representative sample displayed in Figure 4, with indicated intensity scales. *(C)* MALDI MSI relative quantification of the additional metabolites indicated in Figure 3C. *(D)* Quantification of metabolite spatial distribution for DHA and ARA from blood vessel to adjacent blood vessel by showing the metabolite intensity as a function of distance between two vessels for three additional tissue areas to Figure 3F. Vasculature position is indicated in red.
