# Supplementary material for: Spatially resolved characterization of tissue metabolic compartments in fasted and high-fat diet livers
Source: PLoS One. 2022 Sep 6;17(9):e0261803. doi: 10.1371/journal.pone.0261803 (PMC9447892; doi:10.1371/journal.pone.0261803)
Supplement: S6 Fig — (PDF) [file pone.0261803.s006.pdf]

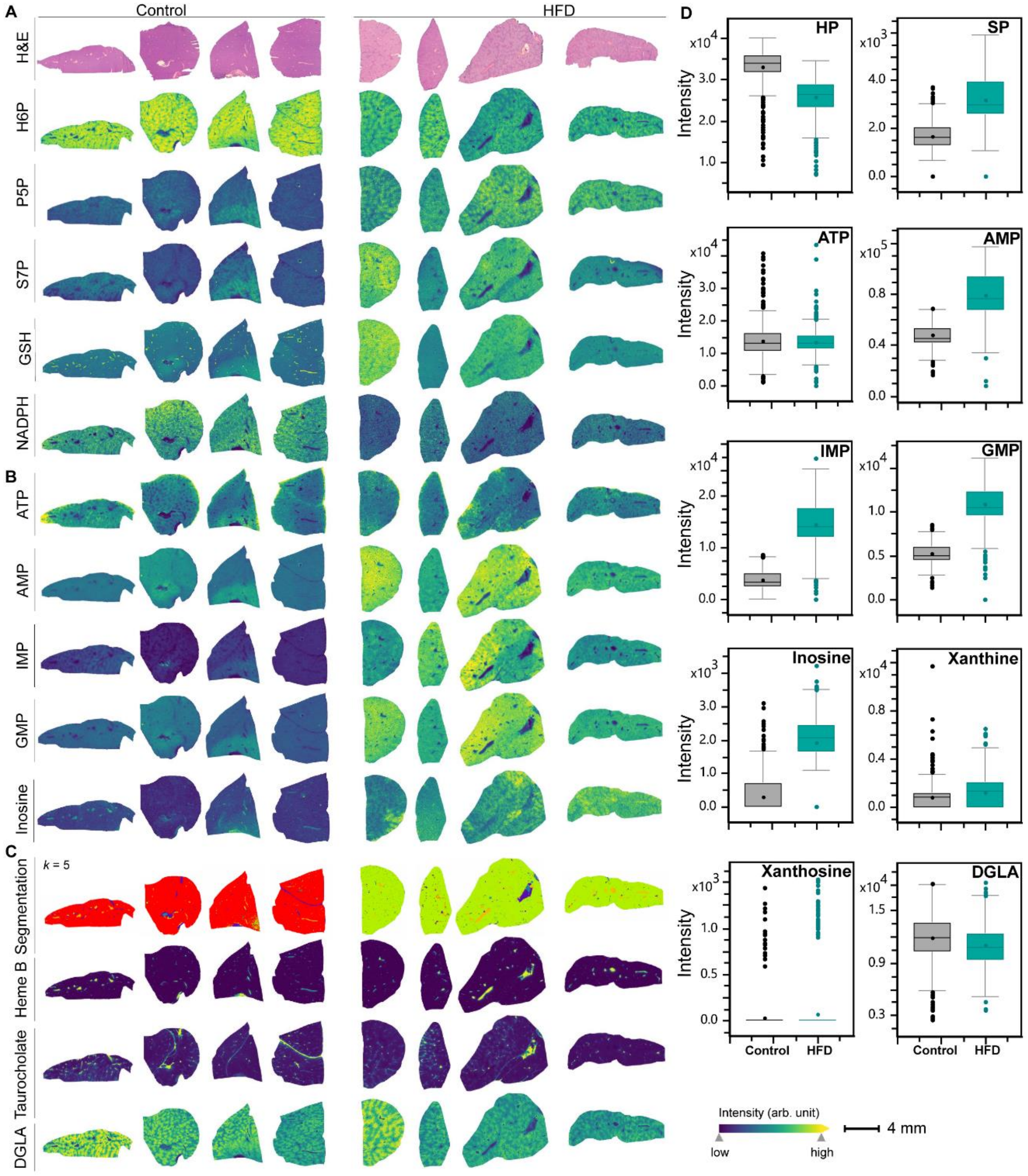

A
Control
HFD
H&E
H6P
P5P
S7P
GSH
NADPH
B
ATP
AMP
IMP
GMP
Inosine
C
k = 5
Segmentation
Heme B
Taurocholate
DGLA
D
HP
SP
ATP
AMP
IMP
GMP
Inosine
Xanthine
Xanthosine
DGLA
Intensity
Control
HFD
Intensity (arb. unit)
low
high
4 mm

77

Supplementary Figure 6. Fatty livers face oxidative stress and increase purine metabolism in response to prolonged nutrient excess. (*C*) H&E optical and MALDI MSI ion images of serial tissue sections from *ad lib* fed mice on a control or high fat diet for 4.5 months (n=5 per group, 2 independent experiments) with the corresponding ion images of the indicated metabolites involved in glycolysis and the pentose phosphate pathway or *(B)* purine metabolims. *(C)* Segmentation map of the MALDI MSI data based on bisecting k-means clustering (k = 5), where each cluster is represented as an individual color, and MALDI MSI ion images of heme B as a marker of the vasculature corresponding to the red segment, taurocholate as a marker of the bile acids corresponding to the purple segment, and DGLA as a representative metabolite that displays a high degree of metabolic zonation. *(D)* MALDI MSI relative quantification of the additional metabolites in Figure 4.
