# Supplementary material for: Spatially resolved characterization of tissue metabolic compartments in fasted and high-fat diet livers
Source: PLoS One. 2022 Sep 6;17(9):e0261803. doi: 10.1371/journal.pone.0261803 (PMC9447892; doi:10.1371/journal.pone.0261803)
Supplement: S1 Table — (PDF) [file pone.0261803.s007.pdf]

Supplementary Table 1, Metabolite annotations.						
**Molecule**	**Chemical formula**	**Theoretical m/z**	**Observed m/z**	**Error (ppm)**	**MSMS peaks**	**MSI Level**
[Fumaric acid-H]$^-$	C4H4O4	115.004	115.004	-0.2		3
[Taurine-H]$^-$	C2H7NO3S	124.007	124.007	1.6		3
[Pyroglutamic acid-H]$^-$	C5H7NO3	128.035	128.035	0.1		3
[Itaconic acid/Glutaconic acid-H]$^-$	C5H6O4	129.019	129.020	-2.1		3
[Creatine-H]$^-$	C4H9N3O2	130.062	130.062	-1.5		3
[Ornithine-H]$^-$	C5H12N2O2	131.083	131.083	-3.0		3
[Malic acid-H]$^-$	C4H6O5	133.014	133.015	-4.9		3
[Adenine-H]$^-$	C5H5N5	134.047	134.047	0.0		3
[Glutamine-H]$^-$	C5H10N2O3	145.059	145.059	-0.3		3
[Xanthine-H]$^-$*	C5H4N4O2	151.026	151.026	2.0	108	2
[Phosphoenolpyruvate-H]$^-$*	C3H5O6P	166.975	166.975	0.6	79	2
[Urate-H]$^-$*	C5H4N4O3	167.021	167.020	4.8	96.020, 97.004, 124.014	2
[DHAP/G3P-H]$^-$*	C3H7O6P	168.991	168.991	0.0	78.954, 96.970	2
[Acetyaspartic acid-H]$^-$	C6H9NO5	174.041	174.041	0.6		3
[Hexose-H]$^-$*	C6H12O6	179.056	179.056	1.7	101.022, 107.085, 113.023, 131.034, 143.046, 149.046, 161.043, 179.055	2
[Phosphoglycerate-H]$^-$*	C3H7O7P	184.986	184.986	1.1	78.960, 96.970, 141.06	2
[Erythrose phosphate-H]$^-$*	C4H9O7P	199.001	199.001	1.0	78.960, 96.970, 138.979	2
[Hexose+Cl]$^-$*	C6H12O6	215.033	215.032	5.1	101.025, 107.033 113.025, 131.045, 149.044, 161.044, 179.055	2
[Pentose phosphate-H]$^-$*	C5H11O8P	229.012	229.012	1.3	96.969, 138.979, 168.991, 192.988	2
[Deoxyadenosine-H]$^-$*	C10H13N5O3	250.095	250.094	0.8	107.035, 134.046	2
[Hexadecenoic acid-H]$^-$	C16H30O2	253.217	253.217	0.4		3
[Palmitate-H]$^-$	C16H32O2	255.233	255.232	4.3		3
[Phosphogluconolactone-H]$^-$	C6H11O9P	257.007	257.007	0.8		3
[Hexose phosphate-H]$^-$*	C6H13O9P	259.022	259.021	4.6	96.970, 138.936, 198.903	2

[Bisphosphoglycerate-H]$^{-}$*	C3H8O10P2	264.952	264.953	-3.0	96.970, 166.974	2
[Inosine-H]$^{-}$*	C10H12N4O5	267.074	267.073	0.7	92.928, 108.019, 135.030	2
[Heptadecanoic acid-H]$^{-}$	C17H34O2	269.249	269.247	4.5		3
[Hydroxyhexadecanoic acid-H]$^{-}$	C16H32O3	271.228	271.227	3.6		3
[Phosphogluconate-H]$^{-}$*	C6H13O10P	275.017	275.018	-0.7	78.959, 96.969, 99.008, 129.018, 159.027, 177.040, 198.903, 257.003	2
[Linolenic acid-H]$^{-}$	C18H30O2	277.217	277.216	4.3		3
[Linoleic acid-H]$^{-}$	C18H32O2	279.233	279.233	-0.7		3
[Oleic acid-H]$^{-}$	C18H34O2	281.249	281.249	0.0		3
[Xanthosine-H]$^{-}$*	C10H12N4O6	283.068	283.069	-1.8	151	2
[Stearic acid-H]$^{-}$	C18H36O2	283.264	283.264	-0.4		3
[Sedoheptulose phosphate-H]$^{-}$*	C7H15O10P	289.033	289.034	-2.4	96.969, 138.979, 155.045, 168.975, 198.999, 229.010	2
[Oxooctadecanoic acid-H]$^{-}$	C18H34O3	297.244	297.242	4.4		3
[Hydroxyoctadecanoic acid-H]$^{-}$	C18H36O3	299.259	299.258	4.2		3
[Arachidonic acid-H]$^{-}$	C20H32O2	303.233	303.232	4.0		3
[Dihomo-linolenic acid-H]$^{-}$	C20H34O2	305.249	305.247	4.3		3
[GSH-H]$^{-}$*	C10H17N3O6S	306.077	306.076	1.0	99.056, 128.035, 143.045, 160.006, 166.097, 177.032, 179.045, 185.055, 197.055, 210.087, 228.096, 231.044, 254.076, 272.086, 288.063	2
[Eicosadienoic acid-H]$^{-}$	C20H36O2	307.264	307.264	2.5		3
[Eicosenoic acid-H]$^{-}$	C20H38O2	309.280	309.280	-0.6		3
[UMP-H]$^{-}$	C9H13N2O9P	323.029	323.028	1.8		3
[Docosahexaenoic acid-H]$^{-}$	C22H32O2	327.233	327.233	0.0		3
[Cyclic AMP-H]$^{-}$	C10H12N5O6P	328.045	328.044	4.1		3
[Deoxyadenosine monophosphate-H]$^{-}$	C10H14N5O6P	330.061	330.060	3.0		3
[dAMP-H]$^{-}$*	C10H14N5O6P	330.061	330.060	2.1	96.972, 134.045, 195.000	2

[Hexose bisphosphate-H]$^{-}$*	C6H14O12P2	338.989	338.989	-0.9	96.969, 138.978, 150.978, 158.924, 168.990, 176.935, 204.989, 241.010	2
[Cyclic GMP-H]$^{-}$	C10H12N5O7P	344.040	344.040	0.2		3
[AMP-H]$^{-}$*	C10H14N5O7P	346.056	346.056	-1.2	96.969, 134.046, 138.979, 150.979, 192.990, 211.000	2
[IMP-H]$^{-}$*	C10H13N4O8P	347.040	347.039	1.2	96.970, 135.030, 138.979, 150.979, 192.989, 211.000	2
[GMP-H]$^{-}$*	C10H14N5O8P	362.051	362.051	0.6	96.970, 150.042, 210.999	2
[S-Adenosylhomocysteine-H]$^{-}$	C14H20N6O5S	383.114	383.116	-5.4		3
[UDP-H]$^{-}$	C9H14N2O12P2	402.995	402.994	1.8		3
[dADP-H]$^{-}$*	C10H15N5O9P2	410.027	410.026	3.7	134.046, 158.924, 176.966, 274.964	2
[ADP-H]$^{-}$*	C10H15N5O10P2	426.022	426.023	-1.2	96.969, 134.046, 158.924, 174.979, 176.935, 192.989, 214.012, 272.957, 290.968, 328.045, 346.056, 408.022	2
[GDP-H]$^{-}$*	C10H15N5O11P2	442.017	442.017	1.4	133.015, 150.041, 158.924, 174.977, 192.990, 211.000, 230.006, 272.956, 290.966, 344.040, 362.051, 424.01	2
[UTP-H]-	C9H15N2O15P3	482.961	482.962	-2.4		3
[Deoxyinosine triphosphate-H]-	C10H15N4O13P3	490.978	490.977	1.2		3
[ATP-H]$^{-}$*	C10H16N5O13P3	505.989	505.986	5.1	96.970, 134.046, 158.924, 176.935, 238.891, 272.957, 328.045, 408.011, 487.976	2
[Taurocholate-H]$^{-}$*	C26H45NO7S	514.284	514.282	4.3	106.981, 124.007	2
[GTP-H]$^{-}$*	C10H16N5O14P3	521.983	521.983	1.3	158.925, 176.934, 272.956, 424.007, 442.016, 503.970	2
[UDP-hexose-H]$^{-}$	C15H24N2O17P2	565.048	565.046	2.6		3
[GSSG-H]$^{-}$*	C20H32N6O12S2	611.145	611.144	0.8	272.089, 288.069, 306.076, 338.048	2
[Heme B-H]$^{-}$	C34H32O4N4Fe	615.170	615.169	1.5		3
[ADP-ribose cyclic phosphate-H]$^{-}$	C15H22N5O16P3	620.020	620.021	-1.0		3
[NADH-H]$^{-}$*	C21H29N7O14P2	664.118	664.117	1.1	134.045, 158.925, 176.934, 272.955, 299.037, 317,052, 328.047, 335.065, 346.055, 397.020, 408.012	2

$[NADPH-H]^{-}$*	C21H30N7O17P3	744.084	744.084	-0.8	134.046, 158.923, 176.938, 272.956, 290.960, 328.047, 397.018, 408.014, 426.023	2
