# Supplementary material for: Spatially resolved characterization of tissue metabolic compartments in fasted and high-fat diet livers
Source: PLoS One. 2022 Sep 6;17(9):e0261803. doi: 10.1371/journal.pone.0261803 (PMC9447892; doi:10.1371/journal.pone.0261803)
Supplement: S2 Table — (PDF) [file pone.0261803.s008.pdf]

Supplementary Table 2

Pathway name	Match status	p-value	Pathway Impact Score
Biosynthesis of unsaturated fatty acids	7/36	1.5E-07	0
Linoleic acid metabolism	2/5	0.0016	1.00
Glycerophospholipid metabolism	3/36	0.011	0.252
Arachidonic acid metabolism	2/36	0.081	0.333
Taurine and hypotaurine metabolism	1/8	0.10	0
alpha-Linolenic acid metabolism	1/13	0.16	0
Arginine biosynthesis	1/14	0.17	0
Glycerolipid metabolism	1/16	0.19	0.012
Purine metabolism	2/66	0.22	0.186
Citrate cycle (TCA cycle)	1/20	0.24	0.030
Pyruvate metabolism	1/22	0.26	0
Phosphatidylinositol signaling system	1/28	0.31	0.002
Alanine, aspartate and glutamate metabolism	1/28	0.31	0.002
Fatty acid elongation	1/39	0.41	0
Fatty acid degradation	1/39	0.41	0
Tyrosine metabolism	1/42	0.43	0.025
Primary bile acid biosynthesis	1/46	0.46	0.023
Fatty acid biosynthesis	1/47	0.47	0.015
