# Supplementary material for: Spatially resolved characterization of tissue metabolic compartments in fasted and high-fat diet livers
Source: PLoS One. 2022 Sep 6;17(9):e0261803. doi: 10.1371/journal.pone.0261803 (PMC9447892; doi:10.1371/journal.pone.0261803)
Supplement: S3 Table — (PDF) [file pone.0261803.s009.pdf]

Supplementary Table 3

Pathway name	Match status	p-value	Pathway Impact Score
Purine metabolism	12/65	2.70E-04	0.313
Pantothenate and CoA biosynthesis	3/19	0.098	0.275
Pentose phosphate pathway	3/22	0.14	0.180
Pyrimidine metabolism	4/39	0.20	0.136
Phenylalanine, tyrosine and tryptophan biosynthesis	1/4	0.22	0.500
Nicotinate and nicotinamide metabolism	2/15	0.22	0
Linoleic acid metabolism	1/5	0.26	0
Arachidonic acid metabolism	3/36	0.36	0.314
Pyruvate metabolism	2/22	0.38	0
Taurine and hypotaurine metabolism	1/8	0.39	0.429
Vitamin B6 metabolism	1/9	0.42	0.078
Caffeine metabolism	1/10	0.46	0
Phenylalanine metabolism	1/10	0.46	0.357
Glycolysis / Gluconeogenesis	2/26	0.46	0.196
Phosphatidylinositol signaling system	2/28	0.5	0.085
Inositol phosphate metabolism	2/30	0.54	0.093
alpha-Linolenic acid metabolism	1/13	0.55	0
Glycerolipid metabolism	1/16	0.63	0.044
Biosynthesis of unsaturated fatty acids	2/36	0.64	0
Glycerophospholipid metabolism	2/36	0.64	0.175
Pentose and glucuronate interconversions	1/18	0.67	0
Citrate cycle (TCA cycle)	1/20	0.71	0
Tryptophan metabolism	2/41	0.71	0.039
Lysine degradation	1/25	0.79	0.141
Glutathione metabolism	1/28	0.82	0.057
Arginine and proline metabolism	1/38	0.91	0
Primary bile acid biosynthesis	1/46	0.94	0.007

Aminoacyl-tRNA biosynthesis	1/48	0.95	0
Metabolism of xenobiotics by cytochrome P450	1/68	0.99	0.036
Steroid hormone biosynthesis	1/85	0.99	0.029
